# Supplementary material for: Association between oral microbiome and seven types of cancers in East Asian population: a two-sample Mendelian randomization analysis
Source: Front Mol Biosci. 2023 Nov 21;10:1327893. doi: 10.3389/fmolb.2023.1327893 (PMC10702768; doi:10.3389/fmolb.2023.1327893)
Supplement: Supplementary file 1 [file DataSheet1.ZIP › Supplementary Materials/MR plots for tongue/tongue═╝/Prostate cancer/pheno.1228_to_prostate cancer_forest.pdf]

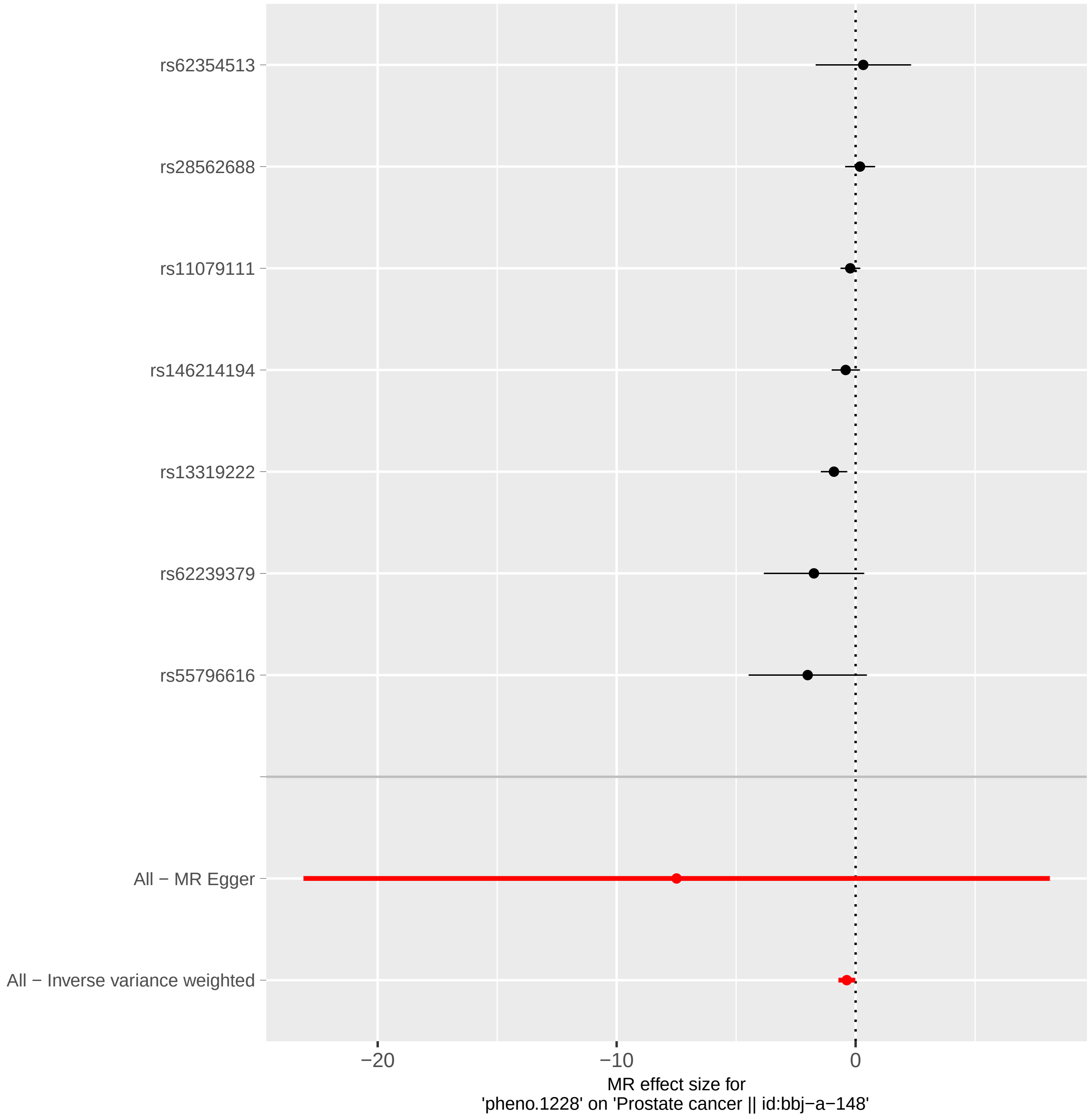

rs62354513
rs28562688
rs11079111
rs146214194
rs13319222
rs62239379
rs55796616
All − MR Egger
All − Inverse variance weighted
−20
−10
0
MR effect size for
'pheno.1228' on 'Prostate cancer || id:bbj−a−148'
